# Histone Arginine Methylation as a Regulator of Gene Expression in the Dehydrating African Clawed Frog (*Xenopus laevis*)

**DOI:** 10.3390/genes15091156

**Published:** 2024-09-01

**Authors:** Saif Rehman, Mackenzie Parent, Kenneth B. Storey

**Affiliations:** Department of Biology, Carleton University, Ottawa, ON K1S 5B6, Canada; saifrehman@cmail.carleton.ca (S.R.);

**Keywords:** epigenetic modifications, dehydration, histone arginine methylation, metabolic rate depression, African clawed frog (*Xenopus laevis*)

## Abstract

The African clawed frog (*Xenopus laevis*) endures prolonged periods of dehydration while estivating underground during the dry season. Epigenetic modifications play crucial roles in regulating gene expression in response to environmental changes. The elucidation of epigenetic changes relevant to survival could serve as a basis for further studies on organ preservation under extreme stress. The current study examined the relative protein levels of key enzymes involved in the arginine methylation of histones in the liver and kidney tissues of control versus dehydrated (35 ± 1%) *X. laevis* through immunoblotting. Protein arginine methyltransferases (PRMT) 4, 5, and 6 showed significant protein level decreases of 35 ± 3%, 71 ± 7%, and 25 ± 5%, respectively, in the liver tissues of the dehydrated frogs relative to controls. In contrast, PRMT7 exhibited an increase of 36 ± 4%. Similarly, the methylated histone markers H3R2m2a, H3R8m2a, and H3R8m2s were downregulated by 34 ± 11%, 15 ± 4%, and 42 ± 12%, respectively, in the livers of dehydrated frogs compared to controls. By contrast, the kidneys of dehydrated frogs showed an upregulation of histone markers. H3R2m2a, H3R8m2a, H3R8m2s, and H4R3m2a were significantly increased by 126 ± 12%, 112 ± 7%, 47 ± 13%, and 13 ± 3%, respectively. These changes can play vital roles in the metabolic reorganization of *X. laevis* during dehydration, and are likely to increase the chances of survival. In turn, the tissue-specific regulation of the histone arginine methylation mechanism suggests the importance of epigenetic regulation in the adaptation of *X. laevis* for whole-body dehydration.

## 1. Introduction

Over time, it has become clear that there is more to molecular biology than simply what is written in DNA. Epigenetic regulatory systems are broadly applied by organisms to enact changes in response to environmental stimuli. Epigenetics refers to modifications to DNA and/or histones that do not change the underlying genetic material but modulate the access of transcriptional machinery to said genetic material. For example, epigenetic mechanisms are emerging as some of the most important contributors to cancer development as well as environmental stress tolerance [1].

Tolerance to environmental stressors such as extreme heat and dehydration is accomplished by animals in a variety of ways that include behavioural, physiological, and molecular adaptations. Past studies have highlighted changes in gene expression as an adaptive response to dehydration [2]. As many of these adaptations occur in a rapidly reversible manner, indicating that epigenetic mechanisms may likely be involved in their regulation. The African clawed frog (*X. laevis*) has long been used as a model organism in developmental biology and cell biology due to its ability to survive extreme environmental conditions. In their native Africa, these frogs are capable of tolerating long seasonal droughts during which they can endure the loss of more than 30% of their body water (due to evaporation across the skin) [3]. The frogs can minimize the loss of body water during the dry season by burrowing into the mud of drying ponds [4], but ultimately, they must rely on physiological and metabolic adaptations to survive. Adaptations that aid their survival include entering a hypometabolic state known as estivation, which is typically characterized by whole body water loss, urea accumulation as a defensive osmolyte, and a state of metabolic rate depression that helps prolong the time that frogs can remain in estivation [5].

When behavioural adaptations fail and water loss rises to high levels, tissue ammonia and urea levels rise, blood viscosity increases and maximal heart rate decreases, leading to vital tissues becoming hypoxic [6,7,8]. In response to limited oxygen supply, metabolic ATP production shifts to favour anaerobic catabolism [9]. Past studies suggest that important glycolytic enzymes, such as pyruvate kinase, are modified to support a necessary increase in anaerobic ATP production [10,11,12,13,14]. Several studies have found potential mechanisms through which these changes can be enacted. Some contributing mechanisms involve changes in signalling cascades and other dynamic reversible regulatory mechanisms such as DNA methylation, histone modification, and miRNA expression [12,15,16,17,18].

Chromatin structure impacts the availability of DNA to transcriptional machinery depending on its conformation. Heterochromatin refers to the densely packed state in which transcriptional efficiency is low, whereas euchromatin refers to the loosely packed state in which transcription is facilitated. Known histone modifications involved in this include acetylation, methylation, ubiquitinoylation, small ubiquitin-like modifier (SUMOylation) and phosphorylation [19,20]. The combined effects of all these modifications working together are commonly referred to as the *Histone code* [21]. Of all these modifications, the most commonly studied are acetylation and methylation that occur on lysine residues in histone tails and are catalyzed by histone acetyltransferases (HATs) and lysine methyltransferases (KMTs), respectively.

Another important modification that is less widely studied is histone arginine methylation. The dysregulation of arginine residue methylation in histone tails has been linked to various cancers as well as metabolic, neurodegenerative, and muscular disorders [22]. Arginine methylation is catalyzed by enzymes known as protein arginine methyltransferases (PRMTs) that make use of S-adenosyl methionine (SAM) as a methyl donor group. There are nine known PRMTs (PRMT1-9) that methylate a wide range of protein targets, including histone tails, transcription factors, translational machinery, signalling molecules, and more [23]. PRMTs are divided into three subtypes based on their catalytic properties. Type 1 PRMTs (PRMT1, 2, 3, 4, 6, and 8) catalyze a reaction resulting in mono-methylarginine (MMA) and asymmetric dimethylarginine (ADMA). Type II PRMTs (PRMT5 and 9) produce MMA and symmetric dimethylarginine (SDMA). The only Type III PRMT is PRMT7 that only catalyzes the formation of MMA [24]. PRMTs have a variety of histone targets which have both activating and suppressing effects on the underlying genes [25,26,27,28,29,30,31,32,33]. Until recently, arginine methylation was thought to be an irreversible modification. However, it is now known that the enzyme peptidyl arginine deaminase 4 (PADI4) converts monomethylated arginine residues into citrulline rather than back to its original unmethylated state [34,35]. Several lysine demethylases (KDMs) can also demethylate arginine residues [36].

The methylation status of arginine residues on histone tails can affect whether the underlying genes are activated or suppressed. Some histone methylation marks are known to silence the underlying genes whereas other marks activate them. PRMT1, 2, 3, and 4 methylate histone tails with marks that have been shown to activate the underlying genes and are therefore known as activating PRMTs [24,27,28,29,37,38]. PRMT5 and 6 deposit marks that are generally repressive to the underlying genes but have also been shown to methylate histones in an activating manner [29,30,31,32,33,39]. PRMT8 is a plasma membrane-associated enzyme that is targeted to the plasma membrane through post-translational myristoylation at the N-terminus [40]. PRMT7 and 9 have several non-histone targets that are involved in cellular processes such as transcription, translation, and cell signalling [23]. Methylated arginine residues can be bound by Tudor domain proteins [41]. The only Tudor domain protein that has been shown to bind methylated arginine residues on histone tails is Tudor domain-containing 3 (TDRD3). The marks deposited by PRMT1 and 4 can be bound by TDRD3 resulting in activation of genes [42].

Histone arginine methylation has been widely studied with respect to disease and development, but the role of this regulatory system in animal adaptation to environmental stress has received little attention to date. The current study was conducted with the goal of determining whether PRMTs play a role in the metabolic reorganization that occurs for frogs to enter estivation. This analysis was conducted by measuring the relative protein levels of PRMT enzymes (1–7) in the livers and kidneys of control vs. highly dehydrated frogs (35 ± 0.93% of total body water lost). In addition, some of the known methylated histone residues (H3R2m2a, H3R8m2a, H3R8m2s, H3R26m2a, H4R3m2a, and H4R3m2s) were also evaluated to help provide a comprehensive assessment of the roles of histone modifications in protecting the genome under dry environmental conditions.

## 2. Materials and Methods

### 2.1. Animal Treatments and Tissues

Ten male adult African clawed frogs (*X. laevis*) were purchased from the University of Toronto. Upon delivery, the frogs were placed in tanks of dechlorinated water at 22 ± 1 °C for 3 weeks before experiments began. Frogs were fed between 3 and 4 CU Adult Frog diet pellets (PMI Nutrition International, Saint Louis, MS, USA) three times per week and their water was changed after each feeding. The animals were then separated randomly into control and dehydration treatment groups and were not fed again (*n* = 5 per group). The animals in the control group were maintained in the conditions outlined above, whereas the animals in the dehydration group were placed into dry containers at 22 °C and allowed to lose water by evaporation across the skin. These animals were weighed twice daily over the course of several days until the desired water loss was achieved. The percentage of body water loss was calculated using the following equation:

$$\%Body\;water\;loss=\frac{m_{i}-m_{d}}{m_{i}\;\cdot BWC_{i}}$$

where $m_{i}$ represents the initial body mass, $m_{d}$ represents the dehydrated body mass, and $BWC_{i}$ is the initial body water content. The initial body water content was determined to be 0.74 ± 0.002 g H_2_O per g body mass [12]. The body water loss percentage of dehydrated animals was 35 ± 0.93%, the limit of what is tolerable by this species [43]. The animals were euthanized by pithing and the liver and kidney tissues were quickly dissected before being flash frozen in liquid nitrogen and stored at −80 °C until needed. All protocols were approved by the Carleton University Animal Care Committee (protocol #106936) and conformed with the guidelines of the Canadian Council on Animal Care.

### 2.2. Total Protein Extraction

Total protein was extracted from frog liver and kidney tissues from the control and high dehydration sample groups (*n* = 5 independent biological replicates per sample group). The samples were placed in ice-cold 1X Lysis buffer (Cell Signalling, Danvers, MA, USA; Cat #43-040) with 1 mM Na_3_VO_4_, 10 mM NaF, 10 mM β-glycerophosphate, and 10 µL/mL protease inhibitor cocktail (BioShop, Burlington, ON, Canada; PIC002) at a 1:5 *w*/*v* ratio. Samples were homogenized immediately after being added to the buffer using a Polytron homogenizer for 10–30 s. Samples were then incubated on ice for 30 min with vortexing every 10 min. After the incubation, the samples were centrifuged at 10,000× *g* for 30 min at 4 °C. Supernatants were collected and transferred to new tubes. The protein concentration was measured using a Bio-Rad protein assay (Bio-Rad, Singapore; Cat #500-0006) with bovine serum albumin used for a standard curve. Sample protein concentrations were then standardized by through addition of small aliquots of homogenization buffer. The samples were stored at −80 °C until needed.

### 2.3. Electrophoresis

As previously described [16], standardized total protein extracts were mixed 1:1 *v*/*v* with 2× loading buffer (100 mM Tris-HCl, 4% *w*/*v* SDS, 0.2% *w*/*v* bromophenol blue, 10% *v*/*v* 2-mercaptoethanol, and 20% *v*/*v* glycerol). Samples were then boiled for 5 min in a water bath and allowed to cool before being stored at −80 °C until used. For electrophoresis, sample aliquots containing 15, 20, or 40 µg protein (depending on the target being assessed) from both conditions (control and dehydrated) were loaded onto 5% acrylamide upper stacking gels sitting atop resolving gels of 8, 10, 12, or 15% depending on the molecular weight of the target protein (acrylamide: bis-acrylamide ratio 29.2:0.8; *w*:*w*). The first lane was loaded with PiNK Plus Prestained Protein Ladder (Froggabio, Concord, ON, Canada: PM005-0500) or BLUeye Prestained Protein Ladder (Froggabio: PM007-0500) depending on the size of the protein of interest. Electrophoresis was performed at a constant voltage of 180 V at room temperature in Trisglycine running buffer (0.25 M Tris-base, 0.035 M SDS, and 2.45 M glycine).

Subsequently, the proteins were transferred from polyacrylamide gels to polyvinylidene difluoride (PVDF) membranes (immobilon-P transfer membrane, Millipore corp., Bedford, MA, USA) at a constant current of 160 mA at 4 °C for 60 to 120 min depending on the size of the protein of interest. The transfer was performed using a transfer buffer (25 mM Tris (pH 8.5), 192 mM glycine, and 20% *v*/*v* methanol). Washing steps were carried out with TBST (150 mM NaCl, 20 mM Tris pH 7.5, 0.05% Tween-20).

### 2.4. Immunoblotting

Following the transfer, PVDF membranes were washed for 3 × 5 min with shaking at room temperature for all washing steps. Membranes were blocked using 5–10% non-fat milk in TBST for 30 min depending on individual blot cross reactivity. Membranes were washed again prior to incubation with primary antibody (1:1000 *v*:*v* TBST) on a shaking platform overnight at 4 °C (Table A1). After incubation with the primary antibody, blots were washed twice with TBST and incubated with an anti-rabbit secondary antibody (diluted 1:2000 in TBST) for 30 min at 21 °C. Following three washes of 10 min each with TBST, the protein bands were visualized by adding 1.4 mL of enhanced chemiluminescence reagent (Pierce, Los Angeles, CA, USA). The Chemi-Genius Bio-Imaging system and Gene Tools software (version #4.3.8.0) from Syngene, MD, USA were used to visualize the membranes. To normalize protein loading, the Coomassie blue total protein staining method was employed. Total protein on the PVDF membrane was stained for 30 min with Coomassie blue solution (0.25% *w*:*v* Coomassie Brilliant Blue R, 50% *v*:*v* methanol, 7.5% *v*:*v* acetic acid) which was also quantified using the ChemiGenius Bio-Imaging system.

### 2.5. Data Quantification

Protein bands were standardized against Coomassie blue-stained PVDF membranes after imaging [44]. Data for each experimental condition are presented as mean ± standard error of the mean (SEM) from five samples obtained from different animals. The fold change was determined for dehydrated samples assigning the control value as 1. Statistical analysis was conducted using a Student’s *t*-test (*p* < 0.05) [45].

## 3. Results

The relative protein levels of key enzymes and targets of the histone arginine methylation regulatory system were analyzed in control and dehydrated conditions in both the livers and kidneys of *X. laevis*. The analysis included the methyltransferase enzymes (PRMT1-7) and some of their key histone targets (H3R2m2a, H3R8m2a, H3R8m2s, H3R26m2a, H4R3m2a, H4R3m2s).

### 3.1. Protein Arginine Methyl Transferases

**Figure 1:**
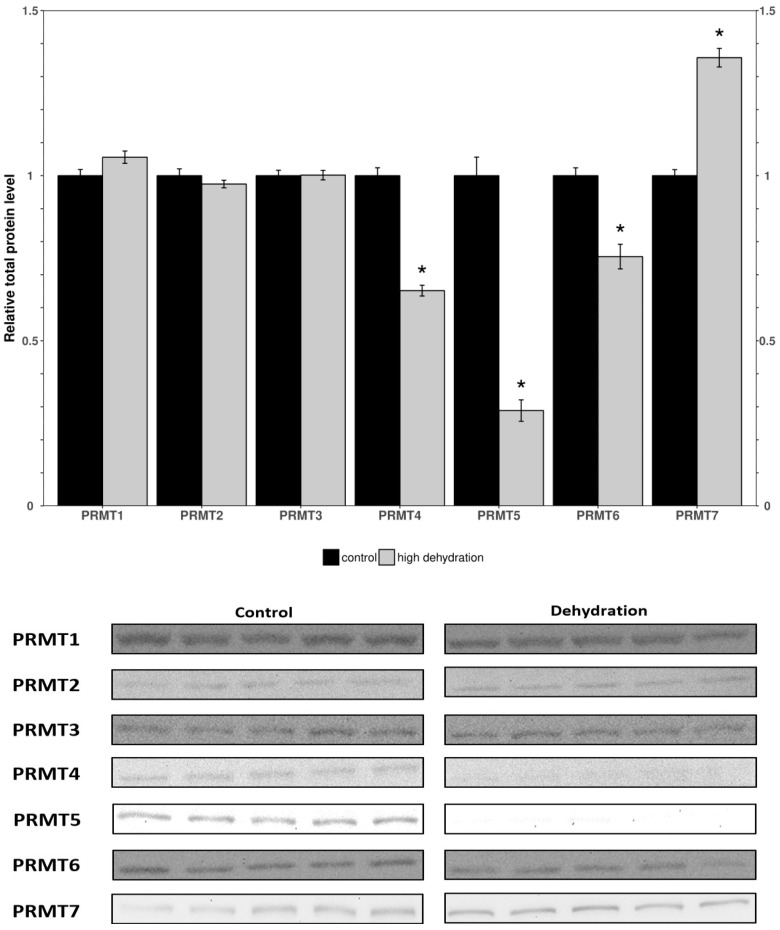
Relative protein levels of PRMTs in the livers of control and dehydrated *X. laevis* as determined by Western blotting. Corresponding bands from immunoblots are shown below the histogram. Data are mean ± SEM, *n* = 5 independent trials on samples from different animals. Data were analyzed using a Student’s *t*-test; asterisks indicate values that are significantly different from the corresponding control (*p* < 0.05).

**Figure 2:**
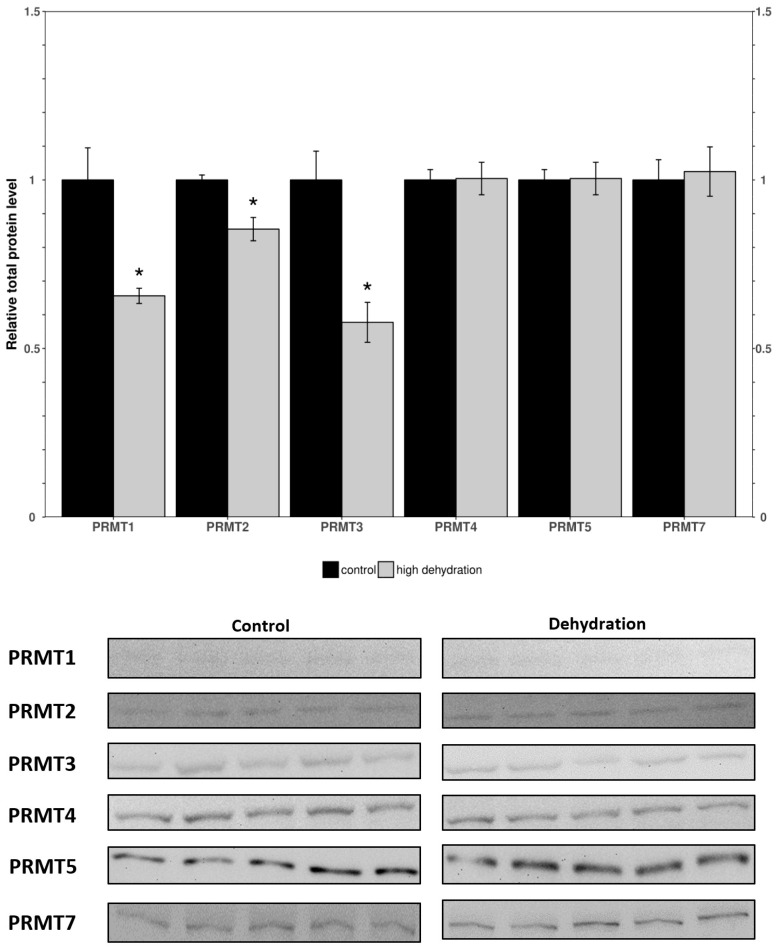
Relative protein levels of PRMTs in the kidneys of control and dehydrated *X. laevis* as determined by Western blotting. Corresponding bands from immunoblots are shown below the histogram. Data are mean ± SEM, *n* = 5 independent trials on samples from different animals. Data were analyzed using a Student’s *t*-test; asterisks indicate values that are significantly different from the corresponding control (*p* < 0.05).

Western blots were performed to analyze the relative protein levels of PRMT1 through 7 in the control and dehydrated conditions of liver and kidney tissues (Figure 1 and Figure 2). In the livers of the dehydrated frogs, PRMT1-3 did not change significantly, whereas the levels of PRMT4, 5, and 6 were all found to decrease: falling by 35 ± 3%, 71 ± 7%, and 25 ± 5%, respectively, relative to controls. Additionally, in the liver, PRMT7 was found to increase by 36 ± 4%. In the kidneys of the dehydrated frogs, PRMT3 was found to increase by 14 ± 3% while all other measured PRMTs (1, 2, 4, 5, 7) did not change significantly relative to the controls. PRMT6 did not cross react in the frogs’ kidneys and therefore was omitted.

### 3.2. Methylated Histone Residues

**Figure 3:**
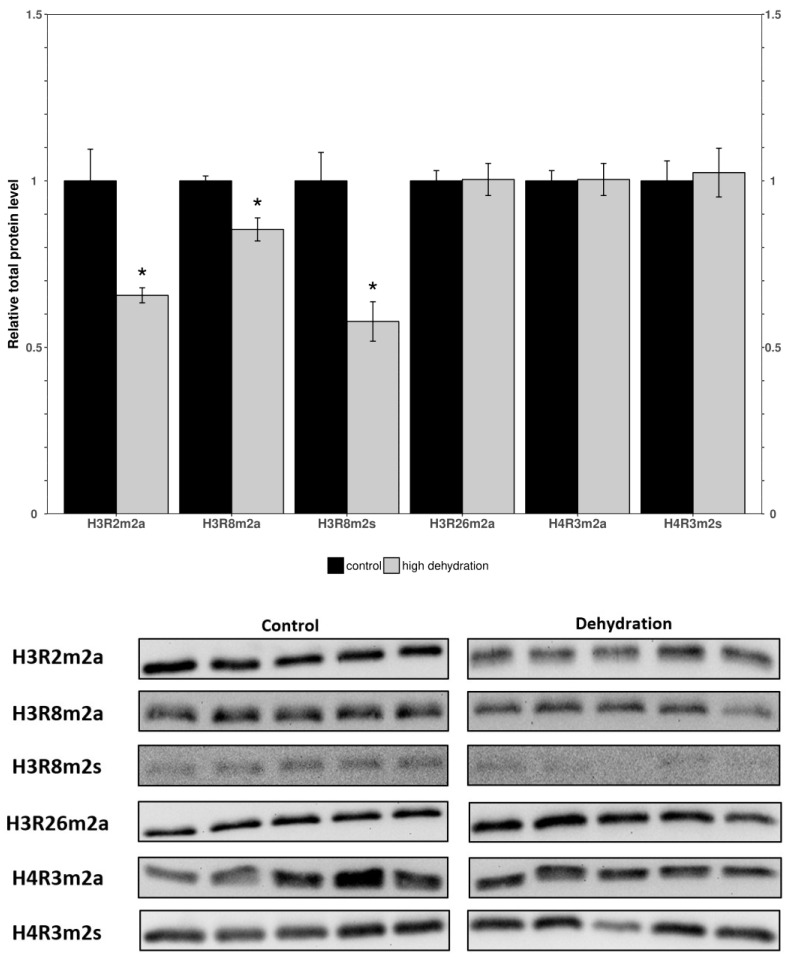
Relative levels of methylated histone residues from the livers of control and dehydrated *X. laevis* as determined by Western blotting. Corresponding bands from immunoblots are shown below the histogram. Data are mean ± SEM, *n* = 5 independent trials on samples from different animals. Data were analyzed using a Student’s *t*-test; asterisks indicate values that are significantly different from the corresponding control (*p* < 0.05).

**Figure 4:**
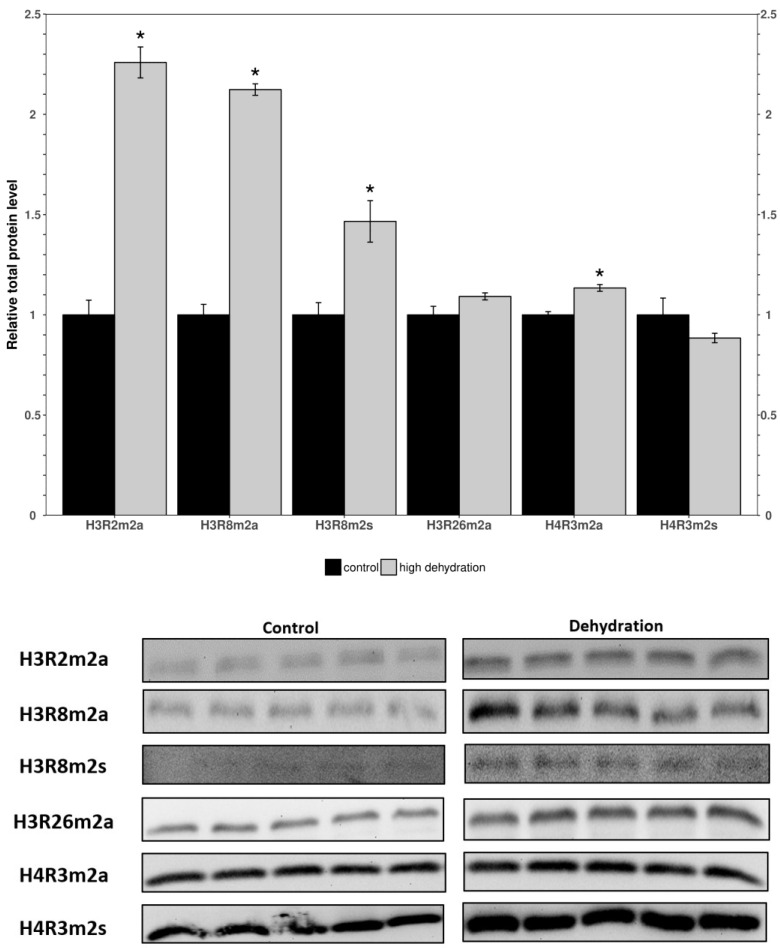
Relative levels of methylated histone residues from the kidneys of control and dehydrated *X. laevis* as determined by Western blotting. Corresponding bands from immunoblots are shown below the histogram. Data are mean ± SEM, *n* = 5 independent trials on samples from different animals. Data were analyzed using a Student’s *t*-test; asterisks indicate values that are significantly different from the corresponding control (*p* < 0.05).

Western blots were performed to determine the relative levels of modified histone residues methylated by PRMTs. The analyzed markers were H3R2m2a, H3R8m2a, H3R8m2s, H3R26m2a, H4R3m2a, and H4R3m2s in the control and dehydrated conditions of the liver and kidney tissues (Figure 3 and Figure 4). In the livers of the dehydrated frogs, H3R2m2a, H3R8m2a, and H3R8m2s, were all found to decrease by 34 ± 11%, 15 ± 4%, and 42 ± 12%, respectively, relative to the controls. In turn, H3R26m2a, H4R3m2a, and H4R3m2s, did not change significantly. In the kidneys of the dehydrated frogs, H3R2m2a, H3R8m2a, H3R8m2s, and H4R3m2a, were all found to increase by 126 ± 12%, 112 ± 7%, 47 ± 13%, and 13 ± 3%, respectively. The other two markers measured in the kidneys, H3R26m2a and H4R3m2s, did not change significantly.

## 4. Discussion

Biological systems face numerous challenges when they experience significant water loss. To cope with such conditions, animals have evolved strategies like estivation. Estivation entails undergoing substantial metabolic modifications that enable organisms to enter prolonged periods of dormancy, often including strategies for body water conservation, while still meeting their energy requirements, with the ability to swiftly revert back to their active state [46]. Various studies have been conducted with the goal of understanding the molecular mechanisms underlying dehydration tolerance in *X. laevis* including investigation of signalling cascades, DNA methylation, histone modifications, and miRNAs [12,15,16,17,18].

To investigate the effects of dehydration on gene expression, organs vital to the stress response, such as the liver and kidneys, were studied. In the livers of dehydrated frogs, PRMTs and histone markers were differentially expressed as compared to the fully hydrated control frogs (Figure 1 and Figure 2). PRMT1, 2, and 3 did not significantly change, whereas PRMT4, 5 and 6 decreased, and PRMT7 increased (Figure 1). There were decreased levels of the methylated histone residues H3R2m2a, H3R8m2a, and H3R8m2s relative to the controls, whereas H3R26m2a, H4R3m2a, and H4R3m2s were not significantly changed (Figure 2). The unchanged H3R26m2a is a known target of PRMT4/CARM1 (co-activator associated arginine methyltransferase 1) that was downregulated. The general decrease in the amount of methylated histone arginine residues is consistent with the downregulation of PRMTs, excluding PRMT7. PRMT4/CARM1 has been shown to deposit transcriptionally activating markers [27,28], whereas PRMT5 and 6 deposit both transcriptionally activating and repressive markers [29,30,31,32,33]. PRMT5 was the most strongly downregulated of the PRMTs in response to dehydration with a 71 ± 7% reduction measured in the livers of the water-stressed frogs (Figure 1). PRMT5 is known to lead to the symmetrical dimethylation of histone H3 and histone H4 (H4R3m2s and H3R8m2s) causing direct binding with DNMT3A and resulting in the DNA methylation of adjacent CpG dinucleotides, which silences transcription [47]. The suppression of PRMT5 may play a role in the metabolic reorganization of *X. laevis* during severe dehydration. Past studies of epigenetic modifications in similar extreme stress-surviving animals corroborate the results found in this study. Histone lysine modifications have been found to experience a similar general reduction in protein levels in *Rana sylvatica* liver tissue during freezing [16]. These changes have been linked in a variety of stress-tolerant species to a decrease in transcriptional activation and the maintenance of a metabolic rate depression (MRD) [17,18]. Incidentally, the upregulation of the type III enzyme, PRMT7, may also be significant for the reorganization of frogs’ metabolisms during dehydration. As well as working in concert with other PRMTs to epigenetically regulate transcription, PRMT7 has many non-histone targets such as transcription initiation factors and heat shock proteins, and its activity on these proteins could be the reason for its upregulation during dehydration [48,49,50]. Interestingly, the methylation of eIF2a by PRMT7 has been shown to regulate the formation of stress granules and is thought to be an important regulator of stress response pathways. These possibilities, potentially explaining the increase in PRMT7 protein levels, must be further explored to reach a definitive conclusion.

When comparing the findings from the liver to kidney tissues, surprising differences in expression were noted. In the kidneys from dehydrated frogs, PRMTs and histone markers were differentially expressed compared to controls (Figure 3 and Figure 4). PRMT enzymes were almost entirely unchanged except for a slight increase in PRMT3 (Figure 3). However, the histone markers were mostly upregulated apart from H3R26m2a and H4R3m2s which did not show significant differences (Figure 4). The large increase in levels of histone markers with little to no change in PRMT levels could indicate that some of the enzymes have undergone post-translational modifications resulting in increased activity. Another possible explanation for the large increase in histone methylation could be a downregulation of histone demethylase enzymes. In either case, the upregulation of these marks suggest that they may play a significant role in the epigenetic reorganization of *X. laevis* metabolism. The H3R2m2a, H3R8m2a, and H3R8m2s markers were all highly upregulated (126 ± 12%, 112 ± 7%, 47 ± 13%; Figure 4). The H3R2m2a and H3R8m2s markers, written by PRMT6 and PRMT5, respectively, are repressive markers that have been shown to downregulate expression of underlying genes [51]. By contrast, the H3R8m2a marker, written by PRMT2, has been shown to activate transcription in underlying genes [38]. The upregulation of these markers (both activating and repressive) supports the idea of the “*Histone code*” that states that the effects of many modifications working in unison determine the availability of genes to transcriptional machinery [21]. Comparable studies of histone lysine modifications conducted in freshwater turtles, which survive extended periods of anoxia, have similarly deduced a dynamic regulation of this mechanism as is necessary for survival [52]. These data support the idea that the reorganization of metabolism in *X. laevis* under dehydrating conditions is not as simple as shutting off genes to save energy. Energetic needs still need to be met and this could involve switching to a greater involvement of anaerobic metabolism that may be needed if reduced blood flow restricts the availability of oxygen to tissues [9,10,14,53].

## 5. Conclusions

The expression levels of proteins involved in histone arginine methylation showed a tissue-specific change with almost opposite trends being observed in the liver and kidneys. Nearly all PRMTs and markers showed a downregulation or no change in the liver (Figure 1 and Figure 2), whereas all PRMTs and markers showed an upregulation or no change in the kidneys (Figure 3 and Figure 4). The exception was PRMT7 which was upregulated in the liver. The strong tissue-specific regulation of arginine histone methylation in *X. laevis* during dehydration stress suggests that this pathway could play an important role in the epigenetic regulation of genes in support of survival during the dry seasons. Some further analyses that would be valuable to better understand this pathway are measurements of the demethylase enzymes responsible for removing the histone markers, as well as activity assays for some of the key PRMT enzymes. A CHiP assay of the studied histone modifications could also further validate which genes are being affected by the aforementioned changes. This study provides an introduction to the epigenetic regulation of genes during the dehydration of *X. laevis*.

## Data Availability

The data that support the findings of this study are available from the corresponding author upon reasonable request.

## Appendix A

**Table A1:** Summary of primary antibody information.

Antibody	Company	Catalogue #
PRMT1	Genetex	GTX128211
PRMT2	Santa Cruz	sc-393254
PRMT3	Genetex	GTX116478
PRMT4	Active Motif	AB_2793205
PRMT5	Abclonal	A2290
PRMT6	Abclonal	A7814
PRMT7	Abclonal	A12159
PRMT8	Novus	NBP1-55401
H3R2m2a	MyBioSource	MBS9402172
H3R8m2s	MyBioSource	MB59607605
H3R8m2a	MyBioSource	MBS9409769
H3R26m2a	Abclonal	A2375
H4R3m2a	Abclonal	A2376
H4R3m2s	MyBioSource	MBS126223
